# MJL-1 is a nuclear envelope protein required for homologous chromosome pairing and regulation of synapsis during meiosis in *C. elegans*

**DOI:** 10.1126/sciadv.add1453

**Published:** 2023-02-08

**Authors:** Hyung Jun Kim, Chenshu Liu, Liangyu Zhang, Abby F. Dernburg

**Affiliations:** ^1^ Department of Molecular and Cell Biology, University of California, Berkeley, Berkeley, CA 94720-3200, USA.; ^2^ Howard Hughes Medical Institute, 4000 Jones Bridge Road, Chevy Chase, MD 20815-6789, USA.; ^3^ California Institute for Quantitative Biosciences (QB3), University of California, Berkeley, Berkeley, CA 94720, USA.; ^4^ Biological Sciences and Engineering Division, Lawrence Berkeley National Laboratory, Berkeley, CA 94720, USA.

## Abstract

Interactions between chromosomes and LINC (linker of nucleoskeleton and cytoskeleton) complexes in the nuclear envelope (NE) promote homolog pairing and synapsis during meiosis. By tethering chromosomes to cytoskeletal motors, these connections lead to processive chromosome movements along the NE. This activity is usually mediated by telomeres, but in the nematode *Caenorhabditis elegans*, special chromosome regions called “pairing centers” (PCs) have acquired this meiotic function. Here, we identify a previously uncharacterized meiosis-specific NE protein, MJL-1 (MAJIN-Like-1), that is essential for interactions between PCs and LINC complexes in *C. elegans*. Mutations in MJL-1 eliminate active chromosome movements during meiosis, resulting in nonhomologous synapsis and impaired homolog pairing. Fission yeast and mice also require NE proteins to connect chromosomes to LINC complexes. Extensive similarities in the molecular architecture of meiotic chromosome-NE attachments across eukaryotes suggest a common origin and/or functions of this architecture during meiosis.

## Teaser

A previously-uncharacterized nuclear envelope protein is required for meiotic chromosome pairing and synapsis in C. elegans.

## INTRODUCTION

Sexual reproduction relies on meiosis, the specialized cell division program that produces haploid gametes. During meiosis, homologous chromosomes must pair, synapse, and undergo crossover recombination to segregate accurately. Upon meiotic entry, each replicated chromosome is assembled into an array of loops anchored to a linear structure known as the chromosome axis (*1*, *2*). Pairing of homologs is gradually stabilized by assembly of a protein matrix, the synaptonemal complex (SC), between axes (*1*–*4*). SCs promote and regulate crossover recombination, which results in chiasmata, physical linkages between homologous chromosomes that persist until segregation and mediate bipolar alignment on the spindle (*3*, *5*, *6*).

Chromosome pairing, synapsis, and recombination are promoted by nuclear envelope (NE)–associated chromosome dynamics during meiosis (*7*–*12*). At meiotic entry, chromosomes become tethered to LINC (linker of nucleoskeleton and cytoskeleton) complexes composed of Sad1/UNC-84 homology (SUN) and Klarsicht/ANC-1/Syne-1 homology (KASH) domain proteins that span the two membranes of the NE (*13*, *14*). Cytoskeletal motors interact with LINC complexes on the cytoplasmic face of the NE, resulting in dramatic chromosome movements during early meiotic prophase (*15*–*17*). This often leads to clustering of chromosome ends near cytoplasmic microtubule-organizing centers (MTOCs) to form a chromosome configuration known as the “meiotic bouquet” (*15*, *18*, *19*). However, in meiocytes that lack focal MTOCs, such as those in the nematode *Caenorhabditis elegans*, clustering of the NE attachment sites is absent or less prominent (*20*, *21*).

In *C. elegans*, specialized regions on each chromosome known as “pairing centers” (PCs) promote homolog pairing and initiate synapsis (*20*, *22*–*25*). Each PC recruits one of four meiosis-specific zinc finger proteins, Zinc finger In Meiosis (ZIM-1), ZIM-2, ZIM-3, or High Incidence of Males (HIM-8), through DNA binding sites present in clusters throughout the PC regions (*26*–*28*). During early meiotic prophase, PCs associate with LINC complexes composed of SUN-1 and ZYG-12 (*29*, *30*). ZYG-12 interacts with cytoplasmic dynein and perhaps other microtubule motors to drive processive movement of chromosomes that promotes pairing and synapsis (*21*, *30*–*32*).

In fission yeast, inner NE proteins Bqt3 and Bqt4 are required for tethering of telomeres to LINC complexes (*33*, *34*). In mouse spermatocytes, the small NE protein MAJIN (membrane-anchored junction protein) connects the meiosis-specific shelterin-binding proteins Telomere Repeat Binding Bouquet Formation Protein 1 (TERB1) and TERB2 to LINC complexes (*35*, *36*). Bqt4 and MAJIN share a similar structure, with a single transmembrane domain near their C termini (*35*, *37*). Homologs of mouse MAJIN have been identified in many metazoans but not in nematodes (*38*).

Here, we report the identification and characterization of a previously uncharacterized meiosis-specific NE protein that is essential for interactions between PCs and LINC complexes in *C. elegans*. On the basis of the functions we have characterized, we named it MJL-1 (MAJIN-Like-1).

## RESULTS

### MJL-1 is a previously uncharacterized, meiosis-specific NE protein

In *C. elegans*, defects in meiosis result in a High incidence of males (Him) phenotype due to nondisjunction of the *X* chromosome (*39*); most meiotic mutants also produce many inviable embryos due to autosomal aneuploidy. We used an established “Green eggs and Him” screen, which exploits an *xol-1p::gfp* reporter that is expressed in *XO* (male) embryos, to identify mutant hermaphrodites with elevated meiotic nondisjunction (*40*). Molecular lesions in the mutants were identified by outcrossing to the CB4856 Hawaiian strain, reisolating homozygous mutants, whole-genome sequencing of their progeny, and computational analysis to identify likely causal mutations (*41*–*43*). Most of the mutations that we identified (50 of 52) were in genes previously shown to be important for meiosis, indicating that such Him screens are nearing saturation. Two mutations were found in previously uncharacterized genes. One of these resulted in a premature stop codon in C17E4.4, which encodes a small protein with a single predicted transmembrane domain. On the basis of previous transcriptome data, this gene is specifically expressed in germline and arcade cells (*44*–*46*).

**Fig. 1.**
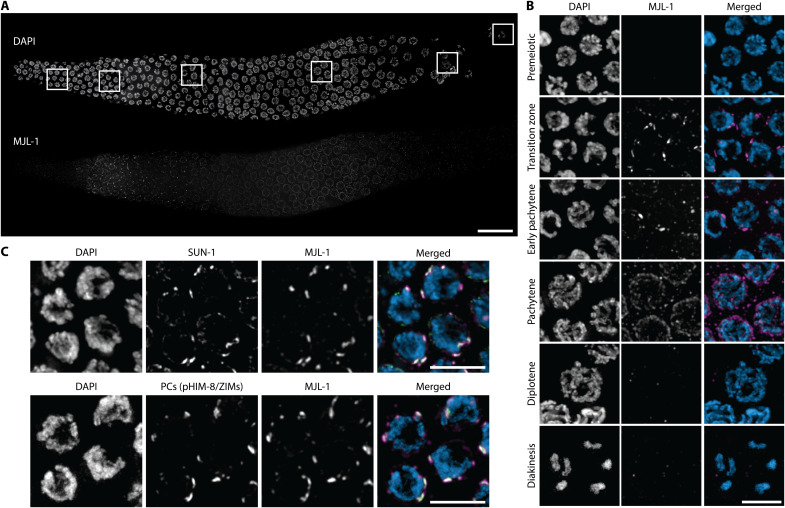
MJL-1 is a meiosis-specific NE protein that associates with PCs and LINC complexes. (**A**) Composite maximum-intensity projection images of whole gonads from *ha::mjl-1* hermaphrodites, stained with 4′,6-diamidino-2-phenylindole (DAPI) (top) and anti-HA antibodies (bottom). Scale bar, 20 μm. (**B**) Examples of nuclei at different stages of meiotic prophase. Scale bar, 5 μm. (**C**) HA-tagged MJL-1 colocalizes with SUN-1 (top) and phosphorylated PC proteins HIM-8 and ZIM-1, ZIM-2, and ZIM-3 (bottom) in early meiotic nuclei. PC proteins were detected using a phospho-specific antibody that recognizes these proteins when phosphorylated by CHK-2 (Checkpoint kinase 2 homolog) (*54*). Scale bars, 5 μm.

Using CRISPR-Cas9–based genome editing, we inserted a hemagglutinin (HA) epitope tag at the N terminus of the C17E4.4 protein. Hermaphrodites homozygous for this insertion produced a normal number of embryos and a slightly elevated frequency of male self-progeny (~1%, compared to 0.2% in wild-type broods). Immunofluorescence of the HA-tagged protein showed NE-specific localization throughout the meiotic region of the germ line. The protein was undetectable in proliferating germline stem cells but was clearly observed at the NE upon meiotic entry and concentrated to form NE “patches” in transition zone (leptotene-zygotene) nuclei. Following synapsis, the protein was redistributed throughout the NE and persisted until late pachytene (Fig. 1, A and B).

During pairing and synapsis, the zinc finger proteins HIM-8, ZIM-1, ZIM-2, and ZIM-3 (which we will refer to as “PC proteins”) bind to PCs and interact with the LINC complex proteins SUN-1 and ZYG-12, which concentrate within the NE to form multiple patches. Immunofluorescence revealed that HA-tagged C17E4.4 colocalized with SUN-1 and all four PC proteins during this transient stage (Fig. 1C).

**Fig. 2.**
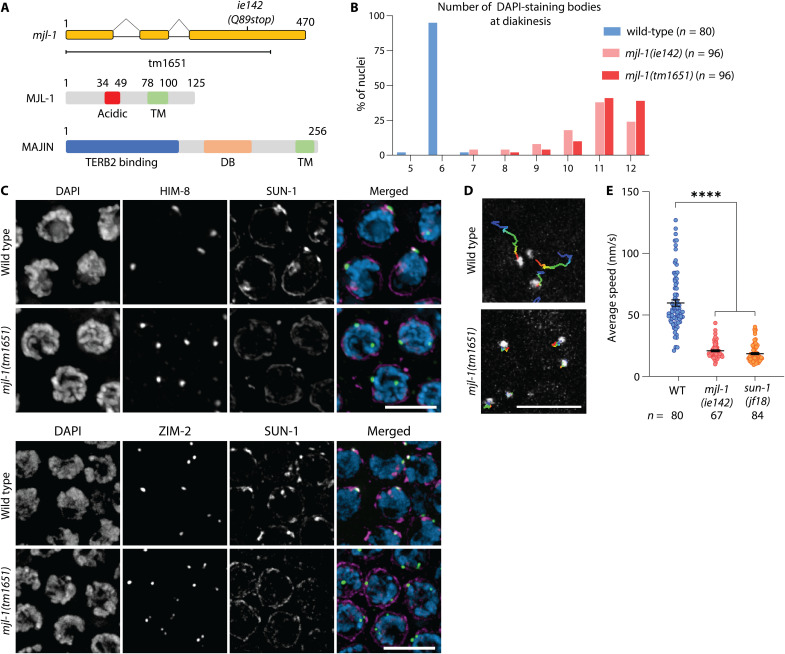
Loss of MJL-1 disrupts PC function. (**A**) Diagram of the *mjl-1* gene, indicating the mutations described in this work (top). Primary structure of MJL-1 in *C. elegans* and MAJIN in *M. musculus* (bottom) (TM, transmembrane; DB, DNA binding domain). (**B**) Number of DAPI-staining bodies in oocyte nuclei at diakinesis in wild-type and *mjl-1* mutant hermaphrodites. (**C**) Loss of MJL-1 disrupts the colocalization of PC proteins and LINC complex. Transition zone nuclei were stained with antibodies against HIM-8 (top) or ZIM-2 (bottom) (green), which mark *X* chromosome or chromosome *V* PCs, respectively, and SUN-1 (magenta). Scale bars, 5 μm. (**D**) Temporal projection of 75-s time course displacement track of GFP::HIM-8 in representative transition zone nuclei in wild type and *mjl-1(ie142)*. Scale bar, 5 μm. (**E**) Average speed of GFP::HIM-8 foci in transition zone nuclei in wild-type (WT), *mjl-1(ie142)* (****P < 0.0001), and *sun-1(jf18)* (****P < 0.0001) hermaphrodites. Each point represents a single nucleus. *P* values were computed using Student’s *t* test with Bonferroni post hoc correction.

On the basis of structural and functional similarities between C17E4.4 and mouse MAJIN (*35*), we named the gene *mjl-1*. Although MJL-1 shares no discernible sequence homology with MAJIN, both are small, single-pass transmembrane proteins with similar meiosis-specific functions (below) (Fig. 2A). MJL-1 is only weakly conserved within the genus *Caenorhabditis*, and we have not yet identified homologs in other nematode genera, including those that express homologs of the PC proteins (fig. S1) (*47*).

### MJL-1 is required for association of PCs with LINC complexes

The mutation identified in our screen, *mjl-1(ie142)*, is likely a null allele because it results in a stop codon before the transmembrane domain. We also obtained a deletion allele, *mjl-1(tm1651)*, from the Japanese National BioResource Project (Fig. 2A). Hermaphrodites homozygous for either mutant allele produced very few viable self-progeny (<1%), and among these, many were males (29 and 33%, statistically indistinguishable), indicative of extensive meiotic nondisjunction. At diakinesis, most oocytes in *mjl-1(ie142)* and *mjl-1(tm1651)* hermaphrodites displayed 10 to 12 4′,6-diamidino-2-phenylindole (DAPI)–staining bodies (Fig. 2B), indicating that chromosomes failed to undergo crossing-over.

We tested whether MJL-1 is required for colocalization of PCs and LINC complexes. Immunofluorescence revealed that in *mjl-1* mutants, SUN-1 did not concentrate with PC proteins or form NE patches in transition zone nuclei (Fig. 2C). However, PC proteins still appeared to associate with the NE, suggesting that they may interact directly with the membrane or another NE protein (fig. S2).

We crossed *mjl-1(ie142)* mutants to a strain expressing green fluorescent protein (GFP)–tagged HIM-8 (*21*) to analyze *X* chromosome movement. The average speed of HIM-8 foci (*X* PCs) in early meiotic nuclei was greatly reduced in the absence of MJL-1, from 59.8 nm/s in wild-type oocytes to 22.7 nm/s in *mjl-1(ie142)*, similar to our measurements for *sun-1(jf18)*, which results in a missense mutation (G311V) near the SUN domain (18.7 nm/s) (Fig. 2, D and E). This agrees with previous analysis of *sun-1(jf18)* (*31*, *32*) and supports the conclusion that the residual chromosome movement in *mjl-1(ie142)* and *sun-1(jf18)* mutants likely results from diffusion rather than active motility (*21*, *32*, *48*). Together, we conclude that the normal interactions between PCs and LINC complexes and the resulting chromosome dynamics are disrupted in *mjl-1* mutants.

### MJL-1 is required to regulate synapsis

**Fig. 3.**
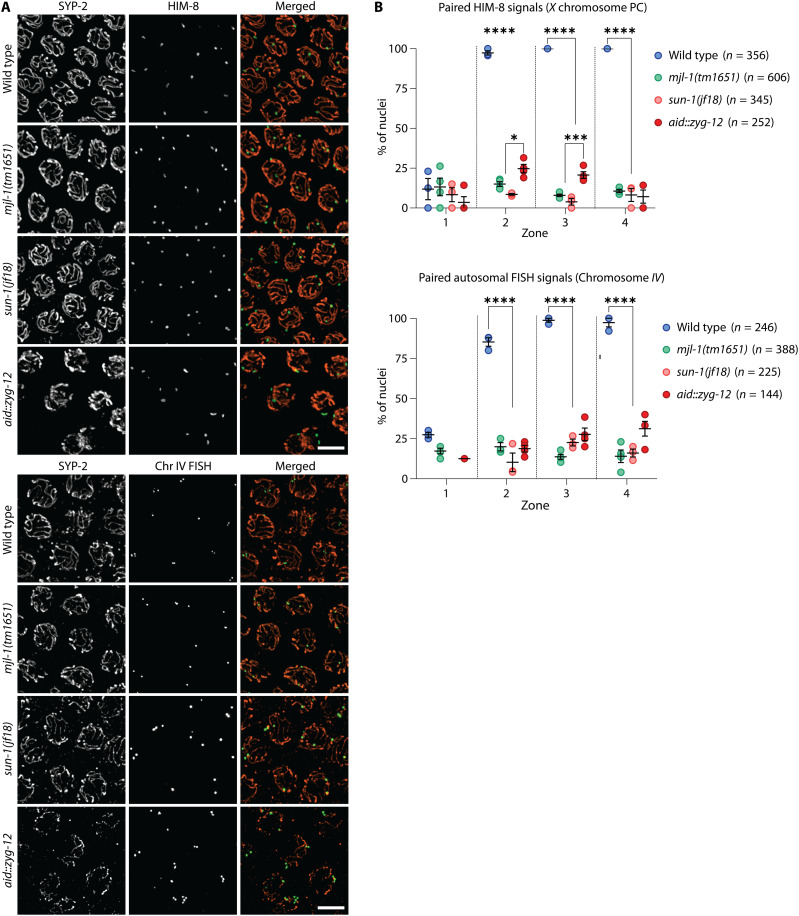
Deletion of *mjl-1* results in promiscuous nonhomologous synapsis. (**A**) Extensive nonhomologous synapsis is observed in *mjl-1(tm1651)* and *sun-1(jf18)* mutants and, to a lesser extent, following depletion of AID::ZYG-12 by treatment with auxin for 12 hours. Mid-pachytene nuclei are stained with antibodies against HIM-8 (green) and SYP-2 (orange). Scale bars, 5 μm. (**B**) Quantification of chromosome pairing in wild-type and mutant hermaphrodites using immunofluorescence (HIM-8) (top) and fluorescence in situ hybridization (FISH) (Chr IV) (bottom) (*P < 0.5; ***P < 0.001; ****P < 0.0001). Gonads were divided into four zones (zone 1, premeiotic cells; zones 2 to 4, region spanning early prophase through pachytene, divided into three zones of equal length). *P* values were calculated by one-way analysis of variance (ANOVA) with pairwise Bonferroni post hoc correction.

Loss of individual PC proteins delays synapsis of the corresponding chromosomes (*26*, *27*). However, in *mjl-1* mutants, we observed extensive SC assembly during early meiotic prophase despite very low levels of homolog pairing. Labeling of specific chromosome loci confirmed that this synapsis occurs between nonhomologous chromosomes (Fig. 3, A and B). Similar promiscuous synapsis was observed in *sun-1(jf18)* mutants and following auxin-induced degradation of ZYG-12 (Fig. 3, A and B), consistent with other evidence that the interaction between PCs and LINC complexes regulates synapsis so that it occurs only between homologous chromosomes (*29*, *30*). How these interactions prevent nonhomologous synapsis remains an open question (*30*).

### MJL-1 depends on SUN-1 for its NE localization

**Fig. 4.**
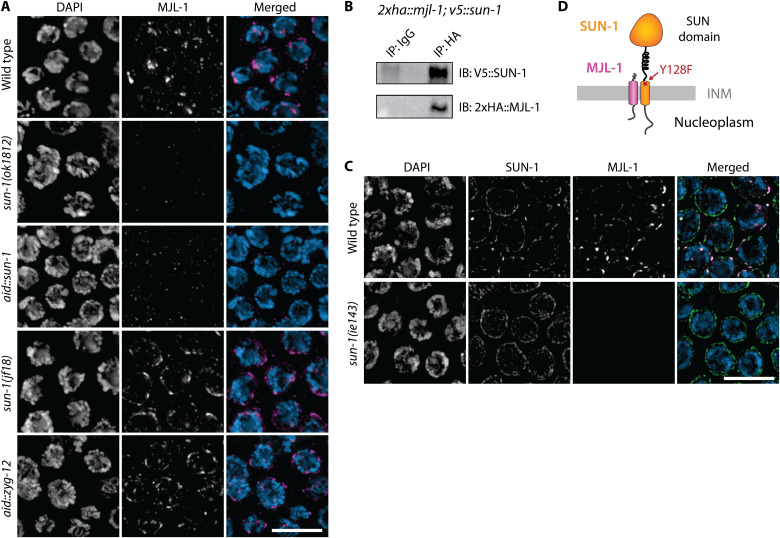
SUN-1 is required for NE localization of MJL-1. (**A**) Transition zone nuclei in wild-type and mutant hermaphrodites expressing HA::MJL-1, stained with anti-HA antibodies (magenta in merged images). Scale bar, 5 μm. (**B**) Coimmunoprecipitation (CoIP) of SUN-1 and MJL-1. HA::MJL-1 was immunoprecipitated using an anti-HA antibody from lysates prepared from *ha::mjl-1*; *v5::sun-1* hermaphrodites. Precipitated proteins were resolved by SDS–polyacrylamide gel electrophoresis and detected using anti-HA and anti-V5 antibodies. IP, immunoprecipitation; IgG, immunoglobulin G; IB, immunoblot. (**C**) The Y128F mutation in *sun-1(ie143)* disrupts interaction between MJL-1 and SUN-1. Transition zone nuclei stained with antibodies against SUN-1 (green in merged images) and HA (magenta) from wild-type and mutant hermaphrodites expressing HA::MJL-1. Scale bar, 5 μm. (**D**) Illustration of the inferred interaction between MJL-1 and SUN-1. INM, inner nucler membrane.

Unexpectedly, we did not detect MJL-1 by immunofluorescence in *sun-1(ok1282)* null mutants or following auxin-induced degradation of SUN-1 (Fig. 4A). The abundance of MJL-1 detected on Western blots was also strongly reduced following auxin-induced degradation of SUN-1 (fig. S3). These findings indicate that SUN-1 is required for NE localization and/or stabilization of MJL-1. In contrast, neither the *sun-1(jf18)* missense mutation nor auxin-induced degradation of the KASH domain protein ZYG-12 disrupted the NE localization of MJL-1 (Fig. 4A), indicating that neither chromosome movement nor ZYG-12 is required for MJL-1 localization and, by extension, for association between MJL-1 and SUN-1. Coimmunoprecipitation of SUN-1 with MJL-1 further supports the idea that these proteins directly interact with each other (Fig. 4B).

Our genetic screen also identified a separation-of-function mutation in *sun-1* that resulted in meiotic defects similar to *sun-1(jf18)* without disrupting the mitotic functions of SUN-1. This missense mutation, *sun-1(ie143)*, changes tyrosine-128 to phenylalanine (Y128F). This conserved tyrosine residue lies near the C terminus of the predicted transmembrane domain in SUN-1 and may contribute to anchoring the transmembrane domain through interactions with phospholipid head groups (*49*). In contrast to *sun-1(jf18)*, *sun-1(ie143)* resulted in loss of MJL-1 protein from the NE (Fig. 4C and fig. S4). This suggests that SUN-1 and MJL-1 may interact through their transmembrane domains and/or adjacent regions in the perinuclear lumen (Fig. 4D). These regions are relatively well conserved within *Caenorhabditis* MJL-1 (fig. S5).

MJL-1 was also detected in apoptotic nuclei in the loop region of the germ line. These nuclei retain SUN-1 but not ZYG-12 at their NE (fig. S6), which may reflect disruption of the outer nuclear membrane during apoptosis, although this has not been directly demonstrated. The persistence of MJL-1 in these nuclei, together with our evidence that the protein requires SUN-1 for its localization to the NE (above) and connects PCs to SUN-1 (below), indicates that MJL-1 probably resides within the inner nuclear membrane with its N-terminal domain in the nucleoplasm, similar to mouse MAJIN (*35*).

### MJL-1 interacts with meiotic PCs and LINC complex proteins

**Fig. 5.**
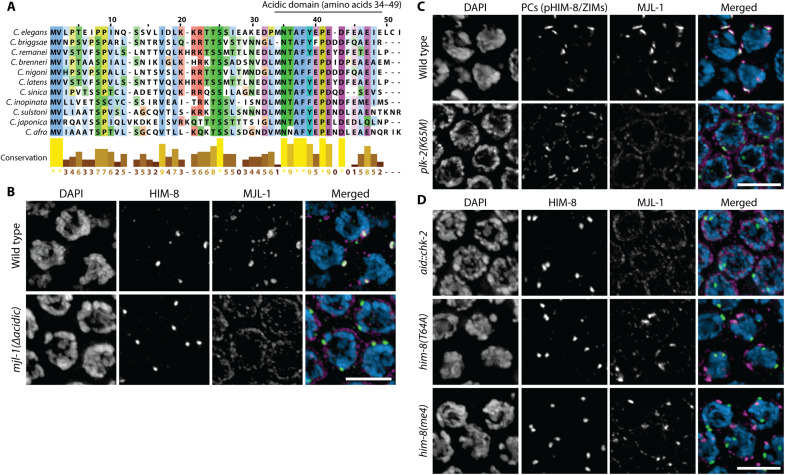
A small domain of MJL-1 enriched in acidic amino acids is essential for its interaction with PC proteins. (**A**) Sequence alignment of the N-terminal region of MJL-1 homologs within *Caenorhabditis*, generated using MAFFT. (**B**) Maximum intensity projection images showing transition zone nuclei stained with antibodies against HIM-8 (green in merged images) and HA (magenta) from hermaphrodites expressing HA::MJL-1 (top) or HA::MJL-1^Δacidic^ (bottom). Scale bar, 5 μm. (**C**) PLK-2 activity is required for interaction between MJL-1 (magenta) and PC proteins (green). Scale bar, 5 μm. (**D**) Recruitment of PLK-2 by HIM-8 (green), which is abrogated in *him-8(T64A)* and *him-8(me4)* mutants, is required for the association of HIM-8 with MJL-1 (magenta). Scale bar, 5 μm.

Sequence alignment of *Caenorhabditis* MJL-1 homologs revealed a short region of relatively high conservation containing several acidic residues (Fig. 5A). Using two synthetic CRIPSR RNAs (crRNAs) flanking this region, we generated an in-frame deletion of amino acids 34 to 49. The resulting MJL-1^Δacidic^ protein was expressed and localized to the NE but did not colocalize with PCs (Fig. 5A and fig. S7). Animals homozygous for this deletion allele showed extensive nonhomologous synapsis, similar to *mjl-1* null mutants (fig. S8). Together with evidence that SUN-1 is essential for localization and stability of MJL-1 (above), this suggests that the mutant protein retains the ability to interact with SUN-1 but does not link PC proteins to SUN-1. In contrast, an in-frame deletion of MJL-1 amino acids 9 to 26, which are also fairly well conserved within MJL-1 homologs, led to no apparent defects (fig. S9).

The Polo-like kinase PLK-2 is recruited to PCs through Polo box–binding motifs in the PC proteins and is required for colocalization of PCs and LINC complexes (*50*, *51*). In animals expressing only kinase-dead PLK-2^K65M^ (*52*, *53*), MJL-1 localized throughout the NE and did not concentrate with PC proteins (Fig. 5B). This indicates that PLK-2 activity is likely required for association between PC proteins and MJL-1/SUN-1 but presumably not for interaction between MJL-1 and SUN-1. Recruitment of PLK-2 to PCs depends on phosphorylation of the Polo box–interacting motifs in the HIM-8/ZIM proteins by Checkpoint kinase 2 homolog (CHK-2) (*54*). Consistent with this, we found that auxin-induced degradation of CHK-2 also abrogated the colocalization of MJL-1 and PCs (Fig. 5C).

We examined the colocalization of MJL-1 with two mutant versions of HIM-8, the zinc finger protein specific for the *X* chromosome PC: HIM-8^T64A^, which has a point mutation in its only Polo box–interacting motif (*50*), and HIM-8^S85F^ [encoded by the *him-8(me4)* allele], which is not phosphorylated by CHK-2 and thus does not recruit PLK-2 (*54*). Neither mutant protein colocalized with patches of MJL-1 (Fig. 5C). Together, we conclude that recruitment of PLK-2 to HIM-8 is required for its association with MJL-1 and SUN-1. This is likely to be true for autosomal PCs as well, based on the failure of MJL-1 to form patches in *plk-2(K65M)* homozygotes.

### MJL-1 directly promotes homolog pairing

**Fig. 6.**
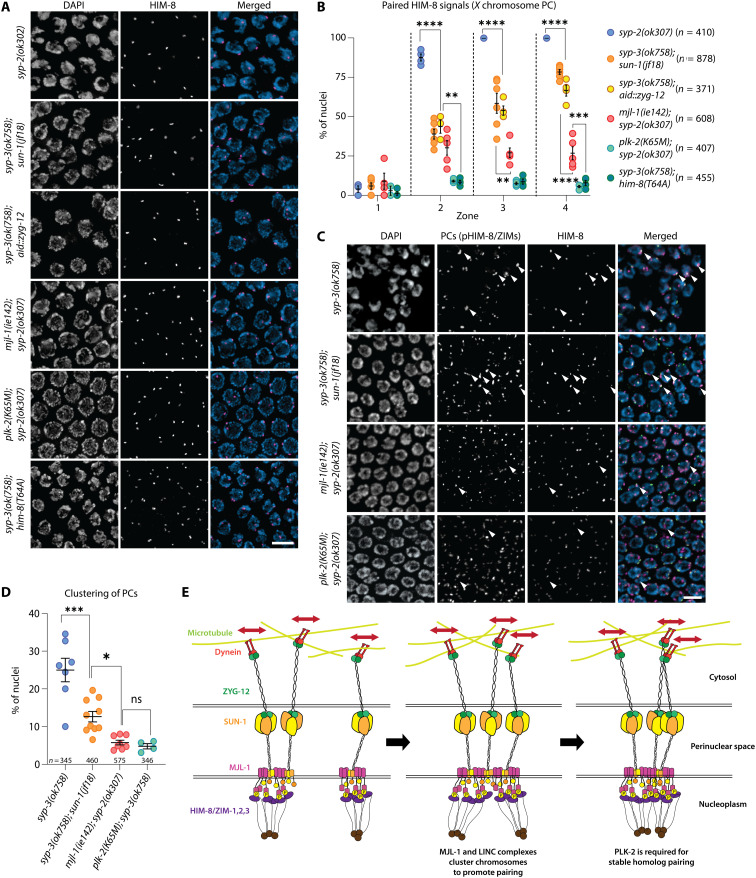
MJL-1 promotes pairing even in the absence of chromosome movements. (**A**) Blocking synapsis does not restore pairing of HIM-8 in *mjl-1(ie142)* mutants, in contrast to *sun-1(jf18)* or depletion of AID::ZYG-12 by treatment with auxin for 24 hours. Prior work has shown that *syp-2* and *syp-3* null mutants have indistinguishable effects on pairing and synapsis (*72*, *73*). Nuclei display polarized morphology due to a cell cycle delay in the absence of synapsis (*54*). Scale bar, 5 μm. (**B**) Quantification of *X* chromosome pairing. The extended transition zone was divided into three equal regions (zones 2 to 4) by length (zone 1, premeiotic). Each point represents a single gonad. *P* values were calculated by one-way ANOVA with pairwise post hoc Bonferroni correction (**P < 0.01; ***P < 0.001; ****P < 0.0001). (**C**) In the absence of synapsis, clustering of HIM-8 with other PC proteins is lower in *mjl-1(ie142)* than in *sun-1(jf18)*. Arrowheads indicate clusters of PCs. Scale bar, 5 μm. (**D**) Quantification of clustering between HIM-8 and other PC proteins in various mutants. Only nuclei in zone 2 were analyzed since pHIM-8/ZIM staining becomes undetectable in zones 3 and 4. *P* values were calculated using one-way ANOVA with post hoc pairwise Bonferroni correction (*P < 0.05; ***P < 0.001). ns, not significant. (**E**) Overview of homolog pairing. Upon meiotic entry, PCs recruit PC proteins (purple) and are connected to MJL-1 (magenta) and SUN-1 (yellow/orange). CHK-2 and PLK-2 are required for this association. MJL-1 interacts with SUN-1 through its transmembrane/perinuclear region. SUN-1 trimers interact with ZYG-12 dimers (green) that are connected to dynein (red) to generate processive chromosome movements (red arrows). These movements promote homolog searching (left). MJL-1 and SUN-1 cluster to promote homolog pairing (middle). PLK-2 phosphorylates unknown substrates to stabilize homolog pairing (right).

Mutations in SC proteins prevent nonhomologous synapsis and partially restore *X* chromosome pairing in *sun-1(jf18)* mutants (*30*). To assess the role of MJL-1 in pairing, we compared the extent of *X* chromosome pairing in *aid::zyg-12* following auxin treatment, *sun-1(jf18)*, and *mjl-1(ie142)*, all in the absence of synapsis. Loss of MJL-1 resulted in more severe pairing defects than depletion of ZYG-12 or the *sun-1(jf18)* mutation, indicating that connection of chromosomes to MJL-1 and LINC complexes promotes limited pairing even in the absence of rapid chromosome movements (Fig. 6, A and B). We also observed a reduction in nonhomologous associations between HIM-8 and other PCs in the absence of MJL-1 compared to *sun-1(jf18)* mutants, suggesting that MJL-1 promotes clustering of PCs (Fig. 6, C and D), which may be required for efficient pairing of homologous PCs (Fig. 6E).

### PLK-2 plays multiple roles in homolog pairing

Hermaphrodites expressing kinase-dead PLK-2^K65M^ or the mutant HIM-8^T64A^ protein displayed virtually no *X* chromosome pairing in the absence of synapsis, in contrast to *mjl-1* mutants (Fig. 6, A and B). This indicates that the role of PLK-2 in homolog pairing goes beyond promoting interactions between PCs and the LINC complexes.

Phosphorylation of the nuclear lamin protein LMN-1 by PLK-2 promotes chromosome mobility along the NE during meiosis, likely by partially disrupting the lamina (*52*). To test whether this might account for the essential role of PLK-2 in homolog pairing, we depleted LMN-1 using RNA interference (RNAi). This depletion did not rescue *X* chromosome pairing in *syp-3(ok758)*; *him-8(T64A)* mutants, revealing that PLK-2 activity at PCs contributes to pairing through a lamin-independent mechanism (fig. S10).

In SC-deficient mutants, homologous PCs pair shortly after meiotic entry and dissociate during late prophase (*25*, *55*). We found that dissociation of paired PCs occurs concomitantly with the disappearance of PLK-2, indicating that PLK-2 activity is important to maintain synapsis-independent interactions between homologous PCs (fig. S11).

## DISCUSSION

### Similarities and differences between MJL-1 and MAJIN

We were able to identify homologs of MJL-1 only within *Caenorhabditis*. Homologs of HIM-8 and ZIM proteins can been detected in related nematode genera (*47*) but show rapid divergence, particularly in their N-terminal domains, which act as scaffolds to recruit kinases and may also directly interact with NE proteins. If the zinc finger proteins interact with MJL-1, as suggested by our findings, then rapid coevolution of these proteins may partially account for our inability to detect MJL-1 homologs in other nematode species.

**Fig. 7.**
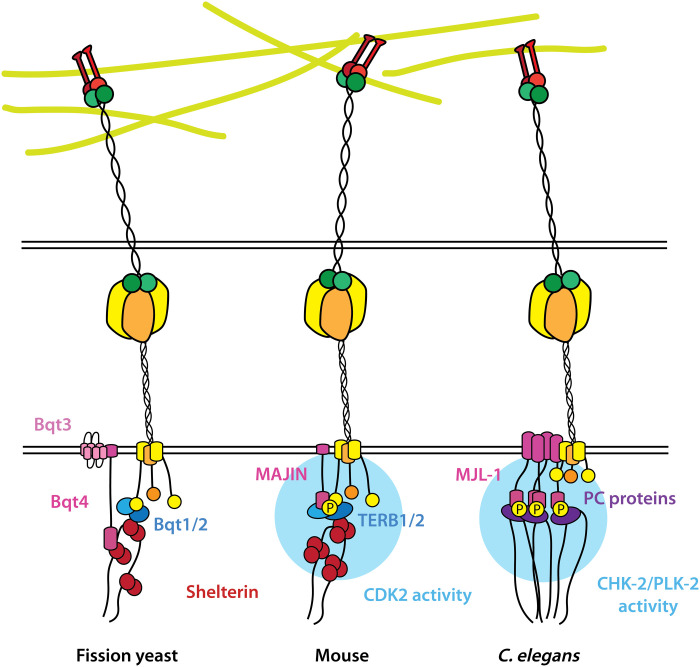
Similarities in molecular architecture of meiotic chromosome-LINC complex attachments in fission yeast, mouse, and *C. elegans*. In fission yeast, Bqt4 (magenta) connects the telomere shelterin complex (red) to the NE in vegetative cells. Bqt4 interacts with a multi-pass NE protein, Bqt3 (pink) (*33*). Meiosis-specific proteins Bqt1 and Bqt2 (blue) are also required to connect telomeres to LINC complexes (yellow/orange) (left) (*33*, *34*). In mice, TERB2 (blue) interacts with MAJIN (magenta), and TERB1 (blue) interacts with SUN-1 (*35*, *56*). MAJIN also associates with SUN-1 (yellow/orange). This requires CDK2 activity (light blue) (middle) (*57*). In *C. elegans*, MJL-1 (magenta) connects PC proteins (purple) to SUN-1 (yellow/orange). This requires CHK-2 and PLK-2 activities (light blue), which are recruited by the PC proteins. MJL-1 and SUN-1 cluster to promote homolog pairing (right).

Our evidence indicates that MJL-1 connects the PC proteins to LINC complexes in *C. elegans*. Similarly, in mice, MAJIN connects shelterin-binding proteins TERB1 and TERB2 to LINC complexes (*35*, *56*). MAJIN interacts with SUN-1 through its nucleoplasmic domain (*57*), while MJL-1 and SUN-1 may interact through their transmembrane domains and/or perinuclear regions. In fission yeast, a direct interaction between NE proteins Bqt3 and Bqt4 and LINC complexes has not been detected (*33*). Whereas MJL-1 requires PLK-2 activity to interact with PC proteins, cyclin-dependent kinase 2 (CDK2) activity is required for interaction between MAJIN and SUN-1 in mice (Fig. 7) (*57*, *58*).

MAJIN and Bqt4 contain N-terminal DNA binding motifs that are required for recruitment of telomeres to the NE (*35*, *37*). The DNA binding motif of MAJIN has been implicated in “telomere cap exchange,” whereby telomeres release shelterin and directly interact with TERB1, TERB2, and MAJIN (*35*). In contrast, MJL-1 lacks an apparent DNA binding motif, and PCs in *C. elegans* appear to associate with the NE even in the absence of MJL-1 or SUN-1. Thus, while MJL-1 shares similarity with MAJIN and Bqt4, the way it connects to chromosomes is likely distinct from vertebrate MAJIN proteins. Together with the lack of apparent sequence homology, this makes it unclear whether MJL-1 is evolutionarily related to other meiotic NE proteins.

### Roles of MJL-1 and LINC complexes in regulation of pairing and synapsis

Connections between PCs and LINC complexes inhibit inappropriate synapsis in *C. elegans* (*29*, *30*). This is consistent with observations that these associations and resulting chromosome movements persist even after pairing of homologous PCs, which occurs soon after entry into meiosis. Loss of telomere-LINC complex connections and/or chromosome movements in mice also leads to some nonhomologous synapsis, albeit not as extensive as in *C. elegans* (*11*, *35*, *56*, *59*). Moreover, loss of CDK2, which is associated with LINC complexes during early prophase in mouse spermatocytes, results in extensive nonhomologous synapsis (*58*, *60*–*62*), suggesting that regulation of synapsis may be a general role of chromosome-LINC complex attachments despite differences in the details of this regulation between organisms. Intriguingly, telomeric attachments in mammals and PC attachments in *C. elegans* each require a kinase (CDK2 and PLK-2, respectively) that is also involved in crossover regulation, suggesting that coordination between chromosome attachment sites and crossover (CO) intermediates may be a conserved feature of meiosis (*63*–*65*).

## MATERIALS AND METHODS

### *C. elegans* strains

N2 Bristol was used as the wild-type *C. elegans* strain; all mutations described here were generated in this background. The Hawaiian isolate CB4856 was used for genetic mapping. All strains were maintained at 20°C under standard laboratory conditions. The following mutations and balancers were used: *mjl-1(ie142) (I:9419844; C to T)*, *mjl-1(tm1651)*, *mjl-1(Δacidic)*, *sun-1(jf18)*, *sun-1(ok1282)*, *sun-1(ie143) (V:13193099; T to A)*, *syp-2(ok307)*, *syp-3(ok758)*, *him-8(me4)*, *him-8(T64A)* (*50*), *plk-2(K65M)::3xflag* (*53*), *nT1[qIs51] (IV;V)*, *hT2 [bli-4(e937) let-?(q782) qIs48] (I;III)*. The following constructs were used for auxin-inducible degradation: *aid::ha::zyg-12* and *aid::v5::sun-1*, and *ha::aid::chk-2*, where “*aid*” designates a DNA sequence encoding a 44–amino acid degron (*66*).

To generate *ha::mjl-1* strains, single-stranded DNA (ssDNA) templates were designed to insert one or two copies of the HA tag (YPYDVPDYA) at the N terminus of MJL-1, separated by a flexible linker (GGGGS). These were coinjected with Cas9-Nuclear Localization Signal (NLS) prebound to duplexed guide RNAs (gRNAs), as well as a gRNA and ssDNA template for co-CRISPR of *dpy-10* (*67*). Final concentrations are as follows: *dpy-10* crRNA, 20 μM; *mjl-1* crRNA, 50 μM; trans-activating CRISPR RNA (tracrRNA), 40 μM; Cas9-NLS protein, 20 μM; *dpy-10* repair template, 1 μM; *mjl-1* repair template, 1 μM. To label MJL-1 using the split-GFP system and V5 tag, a template to insert GFP11 and V5 was coinjected with the Cas9-gRNA ribonucleoprotein complex into DUP223 *glh-1{sam129[glh-1::T2A::sGFP2 (1-10)]}* (*68*). Essentially the same procedure was used to generate in-frame deletions in *mjl-1*, except that two gRNAs were used.

### Auxin-induced degradation

A stock solution containing 250 mM indole acetic acid (auxin) in EtOH was diluted to 1 mM in Nematode Growth Medium) (NGM) agar just before pouring plates. After drying overnight, plates were seeded with *Escherichia coli*: OP50 freshly cultured overnight to saturation in 50 ml of LB was pelleted by centrifugation at 3000*g* for 5 min and resuspended in 500 μl of M9 buffer containing 1 mM auxin. This concentrated bacteria + auxin was spread on the plates and allowed to grow at room temperature for 1 to 2 days. To deplete degron-tagged proteins, young adult animals aged 24 to 48 hours from L4 were picked onto these plates and analyzed 4 to 24 hours later.

### RNA interference

Carbenicillin and isopropyl-β-d-thiogalactopyranoside were added to cooled NGM agar to 200 μg/ml and 1 mM final concentration, respectively, just before pouring plates. Clones from the Ahringer laboratory (*69*) were freshly cultured overnight to saturation in 10 ml of LB containing carbenicillin (200 μg/ml). The culture was pelleted by centrifugation at 3000*g* for 5 min and resuspended in 50 μl of M9 buffer. This concentrated *E. coli* was spread on the plates and allowed to grow at 37°C for 1 day. For feeding RNAi, young adult animals aged 24 to 48 hours from L4 were picked onto these plates and analyzed 24 to 48 hours later. LMN-1 depletion was confirmed by LMN-1 immunofluorescence.

### Cytological methods

Immunofluorescence and in situ hybridization were performed essentially as described previously (*70*). In brief, young adult worms were cut with a scalpel blade in egg buffer containing 0.05% tetramisole to release their gonads on slides, fixed in 1% formaldehyde in egg buffer for 2 min, transferred to tubes, and incubated with methanol prechilled to −30°C for 5 min. The tissue was then washed three times in phosphate-buffered saline (PBS) containing 0.1% Tween 20 (PBST) at room temperature. Tissues were blocked using 1× Roche blocking reagent in PBST for 20 min. Primary antibodies were diluted into the same blocking solution and incubated with the tissues overnight at 4°C. Secondary antibodies were prepared in the blocking solution (1:200), mixed with samples, and incubated 1 to 2 hours at room temperature. Samples were mounted in Prolong Diamond mounting medium containing DAPI (Invitrogen).

For fluorescence in situ hybridization, dissected gonads were fixed in 2% formaldehyde in egg buffer for 5 min, incubated with methanol prechilled to −30°C for 5 min, and washed three times in 2× SSC containing 0.1% Tween 20 (2× SSCT) at room temperature. The tissue was then incubated in 50% formamide in 2× SSCT overnight at 37°C. Fluorophore-conjugated oligonucleotide probes (0.5 to 1 μl of 100 μM; IDT) “IV-2” (*71*) were added to 100 μl of hybridization buffer (50% formamide and 10% dextran sulfate in 2× SSCT), and tissues were moved into this mix and incubated for 2 to 3 min at 91°C and then overnight at 37°C. The tissues were washed three times in 2× SSCT at room temperature and mounted as for immunofluorescence (above).

Images were acquired using a DeltaVision Elite wide-field microscope system (GE) with a 100× 1.40 or 1.45 numerical aperture (NA) oil-immersion objective, or a CSU-W1 SoRa confocal microscope system equipped with a 100×, 1.49 NA oil-immersion objective [Intelligent Imaging Innovations Inc. (3i)]. Deconvolution, projection, and analysis were performed using the softWoRx package or Slidebook 6 (3i).

### Antibodies

All antibodies used in this study were obtained from commercial sources or have been previously described. They include the following antibodies and dilutions: mouse monoclonal anti-HA (Invitrogen 2-2.2.14) (1:250), mouse monoclonal anti-V5 (Invitrogen 46-0705) (1:250), and rabbit polyclonal anti-V5 (Sigma-Aldrich, V8137) (1:250). Custom polyclonal antibodies included rabbit anti–SUN-1 (1:250) (*30*), rat anti–HIM-8 (1:500) (*26*), rabbit anti–SYP-2 (1:500) (*72*), rabbit anti–phospho-HIM-8/ZIMs (1:1000) (*54*), and guinea pig anti–ZIM-2 (1:2000, affinity purified). Fluorophore-conjugated secondary antibodies were purchased from Jackson ImmunoResearch and used at 1:200 dilution.

### Quantification of homolog pairing

Three-dimensional (3D) distances between HIM-8 or fluorescence in situ hybridization (FISH) signals were measured using the “Measure Distance” tool in softWoRx. Foci closer than 0.6 μm were scored as paired. We defined this threshold on the basis of the maximum width of PC protein patches associated with paired chromosomes in wild-type oocytes. For analysis of pairing of FISH signals, we included only nuclei displaying two clear foci.

### In vivo imaging and quantification of chromosome movement

The 3D confocal image acquisition was performed essentially as described (*21*) using a Marianas spinning-disc confocal microscope system equipped with a 100×, 1.46 NA oil-immersion objective [Intelligent Imaging Innovations Inc. (3i)]. Exposure time was set to 100 to 150 ms depending on the brightness of foci. Stacks of 10 to 20 optical sections at 0.5-μm *z* spacing were acquired every 5 s for a total of 5 to 10 min. The 3D time-lapse images were analyzed using Imaris 9.2.0 (Bitplane). Background drift was corrected using the “Reference Frame” tool. Foci were detected using the “Spots” tool with an estimated XY diameter of 1.33 μm and filtered with “Quality” and “Intensity Sum” (Imaris). Tracks were obtained with a max distance of 1.75 μm and a max gap of 3. Tracks from the background noise were manually removed. *P* values were calculated using the pairwise *t* test with post hoc Bonferroni correction.

### Western blots

Two hundred young adult animals aged 48 hours from the L4 stage were picked into tris-buffered saline (TBS) with 0.1% Tween 20 buffer, washed three times, and then incubated in 1× SDS sample buffer at 50°C for 10 min. Samples were vortexed for 2 to 3 min until no visible solid material remained. Proteins were separated using SDS–polyacrylamide gel electrophoresis gradient gels (Invitrogen, NuPAGE, 4 to 12%, bis-tris, 1.0 mm, 10-well, MES SDS running buffer) and transferred to Amersham Hybond P 0.45 polyvinylidene difluoride membranes. The membrane was cut into slices and probed with mouse anti-HA (Invitrogen, 2-2.2.14) (1:1000), mouse anti-V5 (Invitrogen) (1:1000), or mouse anti–α-tubulin (Sigma-Aldrich, DM1A) (1:5000) antibodies overnight at 4°C. Horseradish peroxidase–conjugated donkey anti-mouse secondary antibody (Jackson ImmunoResearch) (1:10,000) was incubated with membranes for 1 hour at room temperature. SuperSignal West Femto Maximum Sensitivity Substrate (Thermo Fisher Scientific) was used for detection. ImageJ mean gray value was used for quantification.

### Immunoprecipitation

Approximately 200,000 young adult animals grown in liquid culture, aged 48 hours from the L4 stage, were collected by centrifugation and homogenized with a Douncer in 1× egg buffer with 250 mM sucrose until 90% of adults were broken. Debris was precipitated by centrifugation at 50*g* for 2 min. The supernatant was filtered using 40-μm filters followed by 20-μm filters to remove additional debris. Nuclei were pelleted by centrifugation at 2000*g* for 10 min and resuspended in 800 μl of lysis buffer [130 mM NaCl, 2.5 mM MgCl_2_, 25 mM Hepes (pH 7.4), 2 mM EGTA, and 1% Triton X-100, with Roche cOmplete Mini EDTA-free protease inhibitor cocktail]. Nuclei were sonicated in a Bioruptor Twin sonication bath (Diagenode, Denville, NJ) at 4°C for 30-min periods of alternating 30 s on high power and 30 s off. The resulting lysate was centrifuged at 10,000*g* for 2 min, and supernatant was incubated with antibody-coated Dynabeads Protein A (Invitrogen) at 4°C for 2 hours and eluted in urea solution (6 M urea, 6% SDS, and 5% 2-mercaptoethanol).

## Notes

View/request a protocol for this paper from *Bio-protocol*.
