# Supplementary material for: MJL-1 is a nuclear envelope protein required for homologous chromosome pairing and regulation of synapsis during meiosis in C. elegans
Source: Sci Adv. 2023 Feb 8;9(6):eadd1453. doi: 10.1126/sciadv.add1453 (PMC9908027; doi:10.1126/sciadv.add1453)
Supplement: Supplementary file 1 — Figs. S1 to S11 Table S1 [file sciadv.add1453_sm.pdf]

Science Advances
AAAS

Supplementary Materials for

MJL-1 is a nuclear envelope protein required for homologous chromosome pairing and regulation of synapsis during meiosis in *C. elegans*

Hyung Jun Kim *et al.*

Corresponding author: Abby F. Dernburg, afdernburg@berkeley.edu

Sci. Adv. **9**, eadd1453 (2023)
DOI: 10.1126/sciadv.add1453

This PDF file includes:

Figs. S1 to S11
Table S1

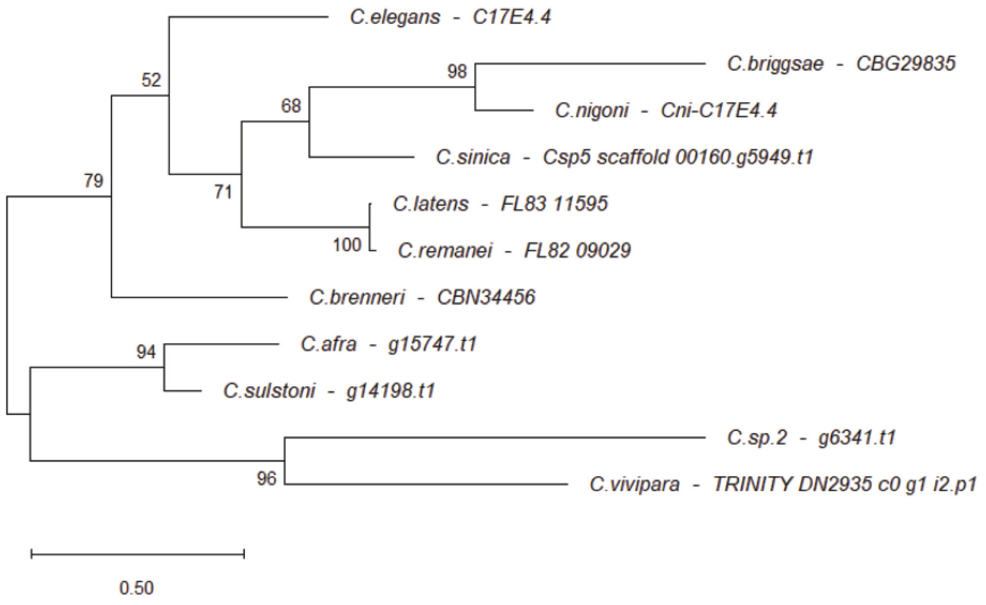

Fig. S1. Phylogenetic tree of MJL-1 homologs in *Caenorhabditis.* A maximum-likelihood phylogenetic tree of MJL-1 homologs from representative *Caenorhabditis* species. Numbers on each node are Bootstrap values. Scale bar, 0.5 substitutions per site.

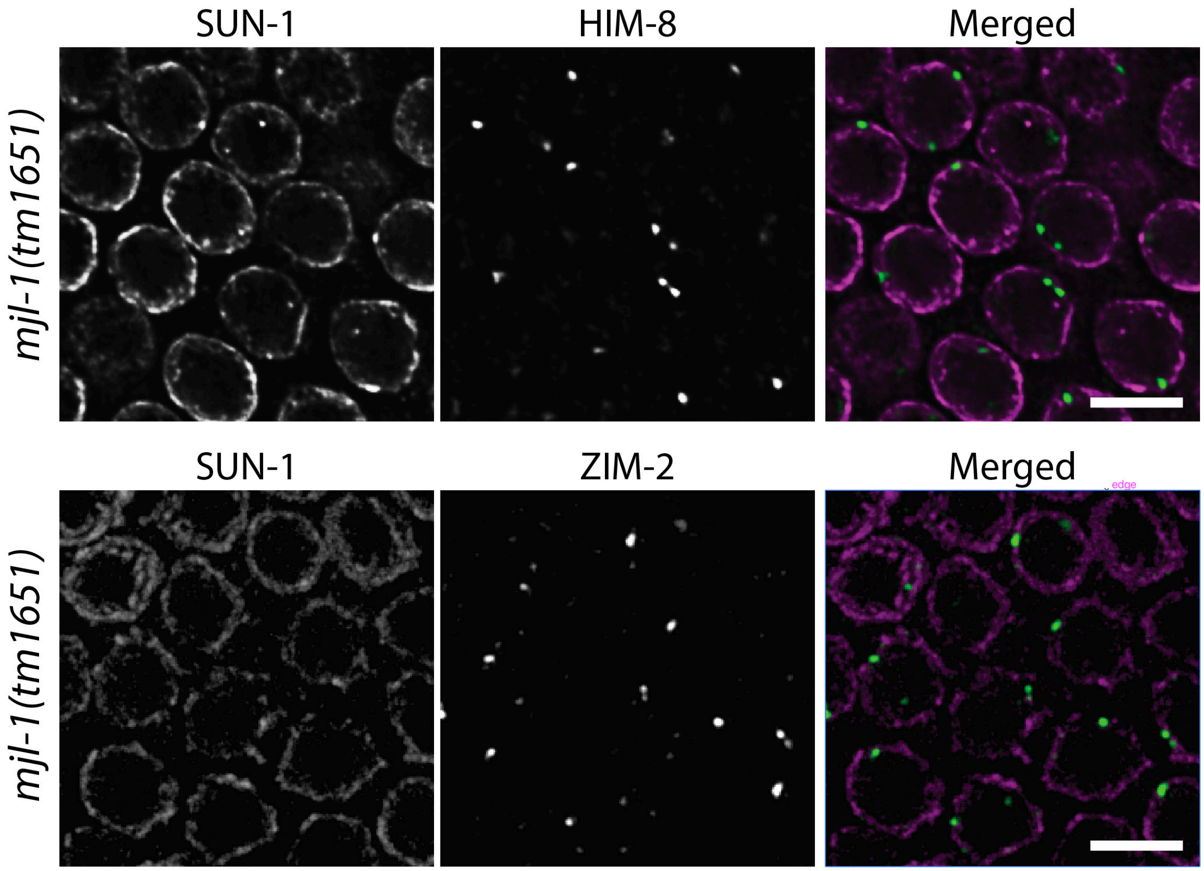

Fig. S2. PCs localize at the NE in the absence of MJL-1. Cross-section images of transition zone nuclei. SUN-1 (magenta) marks the NE in meiotic cells; HIM-8 and ZIM-2 (green) mark *X* chromosome and chromosome *V* PCs, respectively. Although pairing is severely reduced in *mjl-1(tm1615)*, PCs are still associated with the NE. Scale bars, 5 µm.

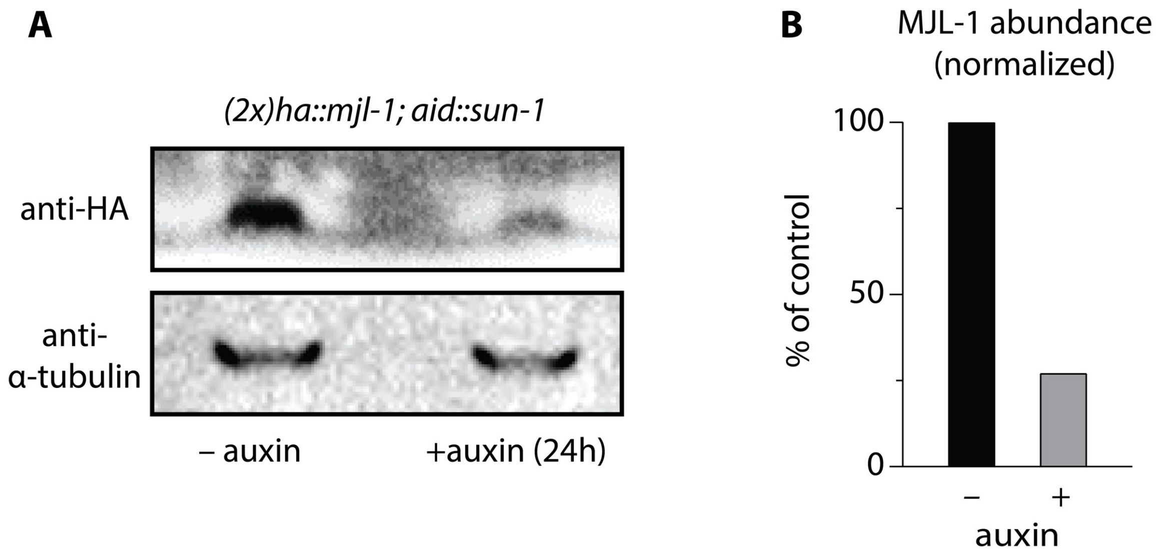

Fig. S3. MJL-1 abundance is greatly reduced in the absence of SUN-1. **(A),** Western blot of proteins in strains expressing 2xHA::MJL-1 and (degron-tagged) AID::SUN-1. MJL-1 was detected with anti-HA antibodies, either in the absence of auxin treatment or following depletion for 24 hours, and normalized by comparison with the α-tubulin band. **(B),** Quantification of (A).

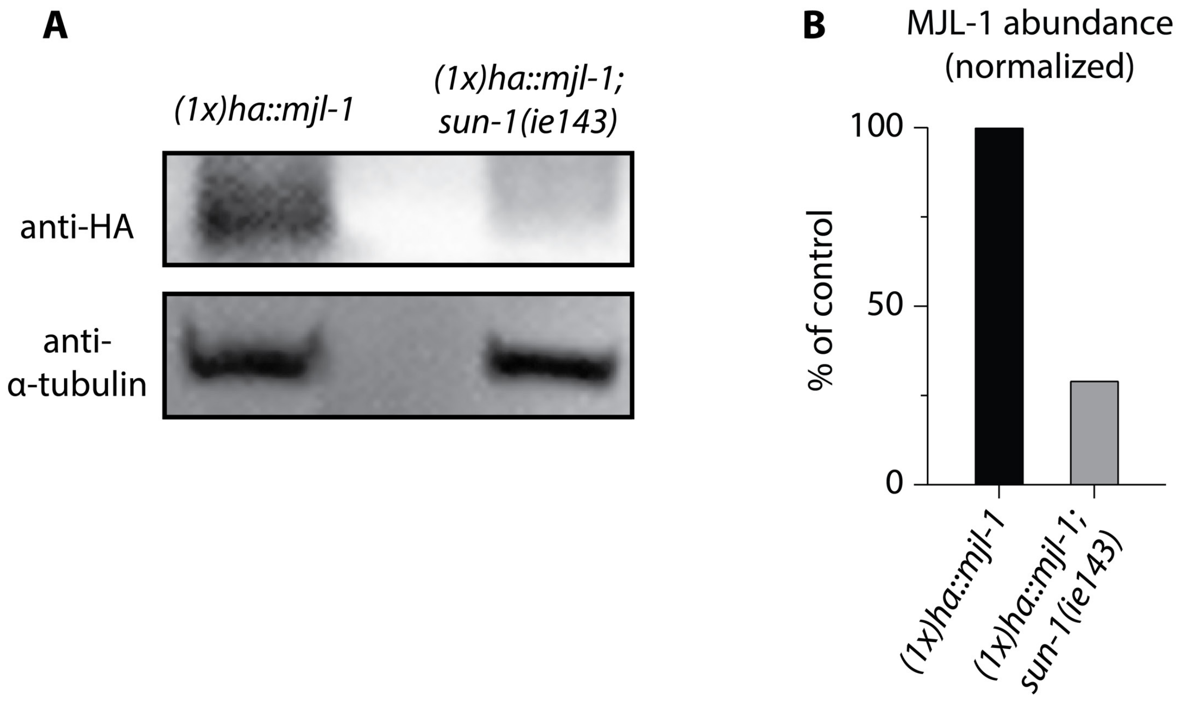

Fig. S4. MJL-1 abundance is greatly reduced in *sun-1(ie143)*. (A), Western blot of proteins in strains expressing 1xHA::MJL-1 with either wild-type *sun-1* or *sun-1(ie143)*. MJL-1 was detected with anti-HA antibodies and normalized by comparison to the intensity of the α-tubulin band. **(B),** Quantification of (A).

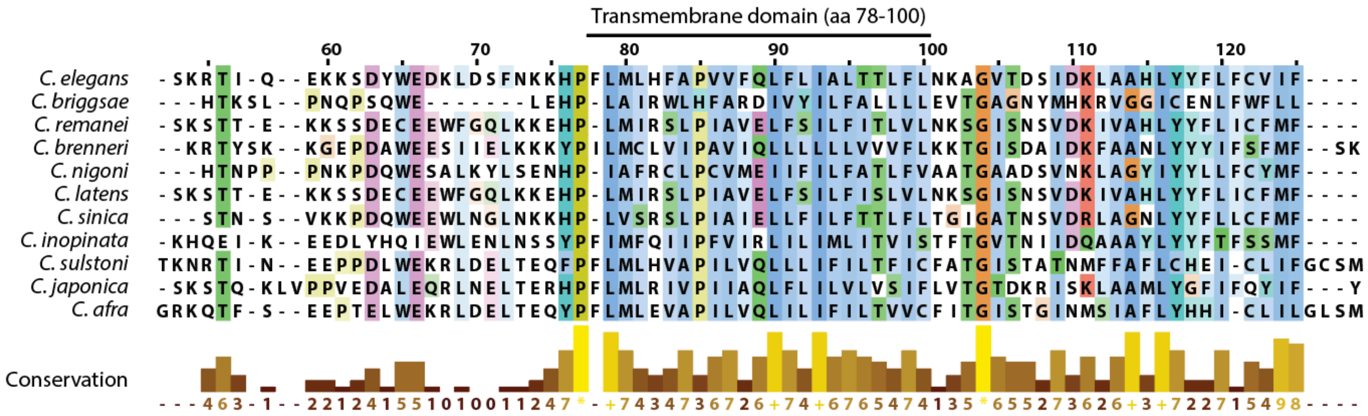

Fig. S5. Sequence alignment of transmembrane and perinuclear regions of C*aenorhabditis* MJL-1 homologs. Alignment was generated using MAFFT.

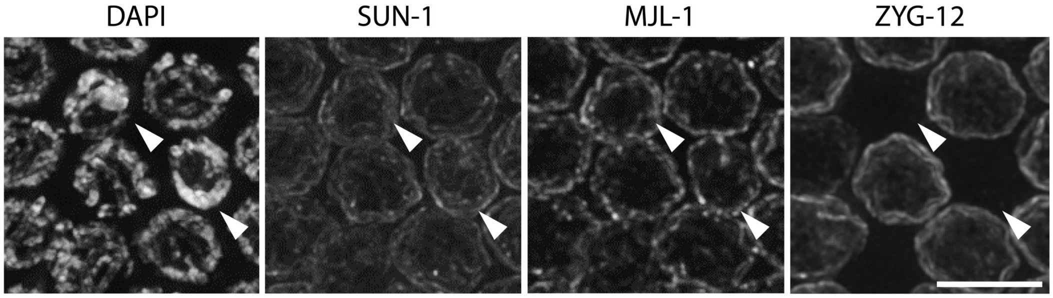

Fig. S6. MJL-1 and SUN-1 are detected at the NE of oocyte nuclei undergoing apoptosis, while ZYG-12 is absent. Maximum-intensity projection images showing late pachytene nuclei. Arrowheads indicate apoptotic nuclei, which show hypercondensed chromatin (bright DAPI staining) and loss of ZYG-12 from the nuclear periphery. Scale bar, 5 µm.

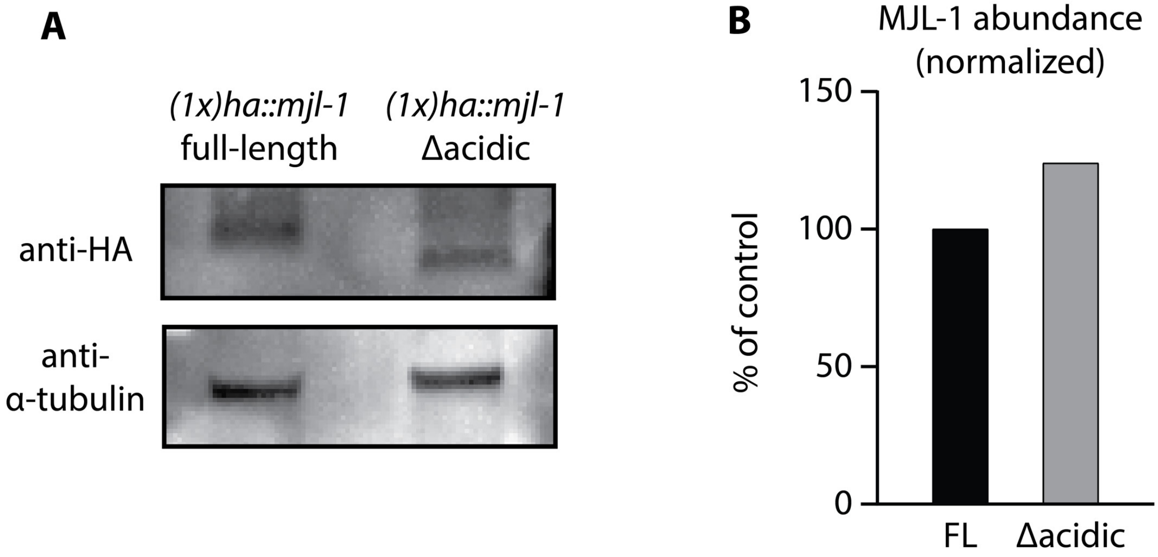

Fig. S7. Deletion of acidic residues does not reduce the stability of MJL-1. (A), Western blot of proteins in strains expressing either 1xHA::MJL-1 or 1xHA::MJL-1$^{\Delta acidic}$. MJL-1 was detected with anti-HA antibodies and quantified in relation to α-tubulin. **(B),** Quantification of (A).

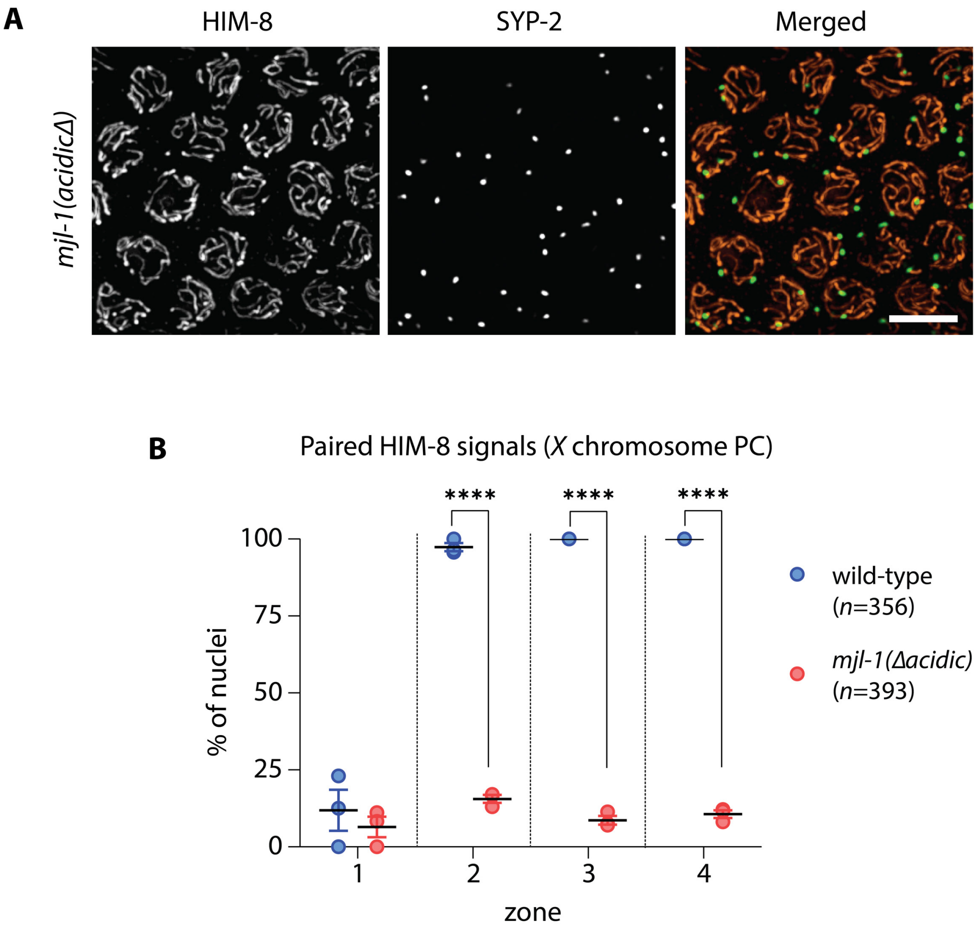

Fig. S8. Deletion of a small acidic region in MJL-1 results in extensive nonhomologous synapsis. (A), Maximum-intensity projection images of pachytene nuclei stained with antibodies against HIM-8 (green) and SYP-2 (orange). Scale Bar, 5 µm. **(B),** Quantification of *X* chromosome pairing in wild-type and *mjl-1(Δacidic)* hermaphrodites (p<0.0001). Each point represents a single gonad. p-values were computed using Student's t-test with Bonferroni *post-hoc* correction.

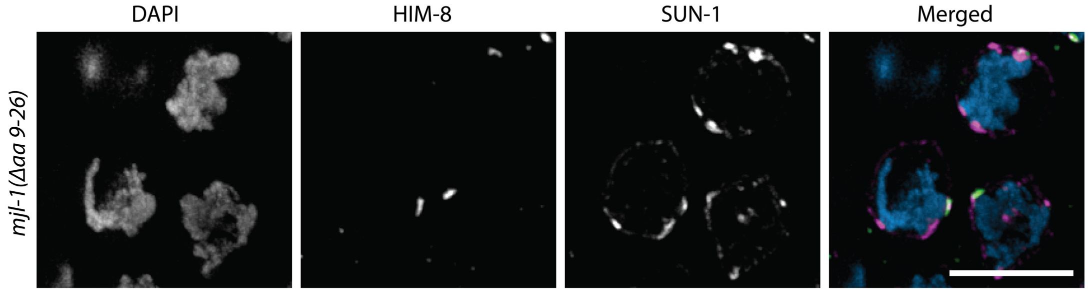

Fig. S9. Deletion of amino acids 9-26 in MJL-1 dose not disrupt the colocalization of HIM-8 and LINC complex. (A), Maximum-intensity projection images of transition zone nuclei stained with antibodies against HIM-8 (green) and SUN-1 (magenta). Scale Bar, 5 μm.

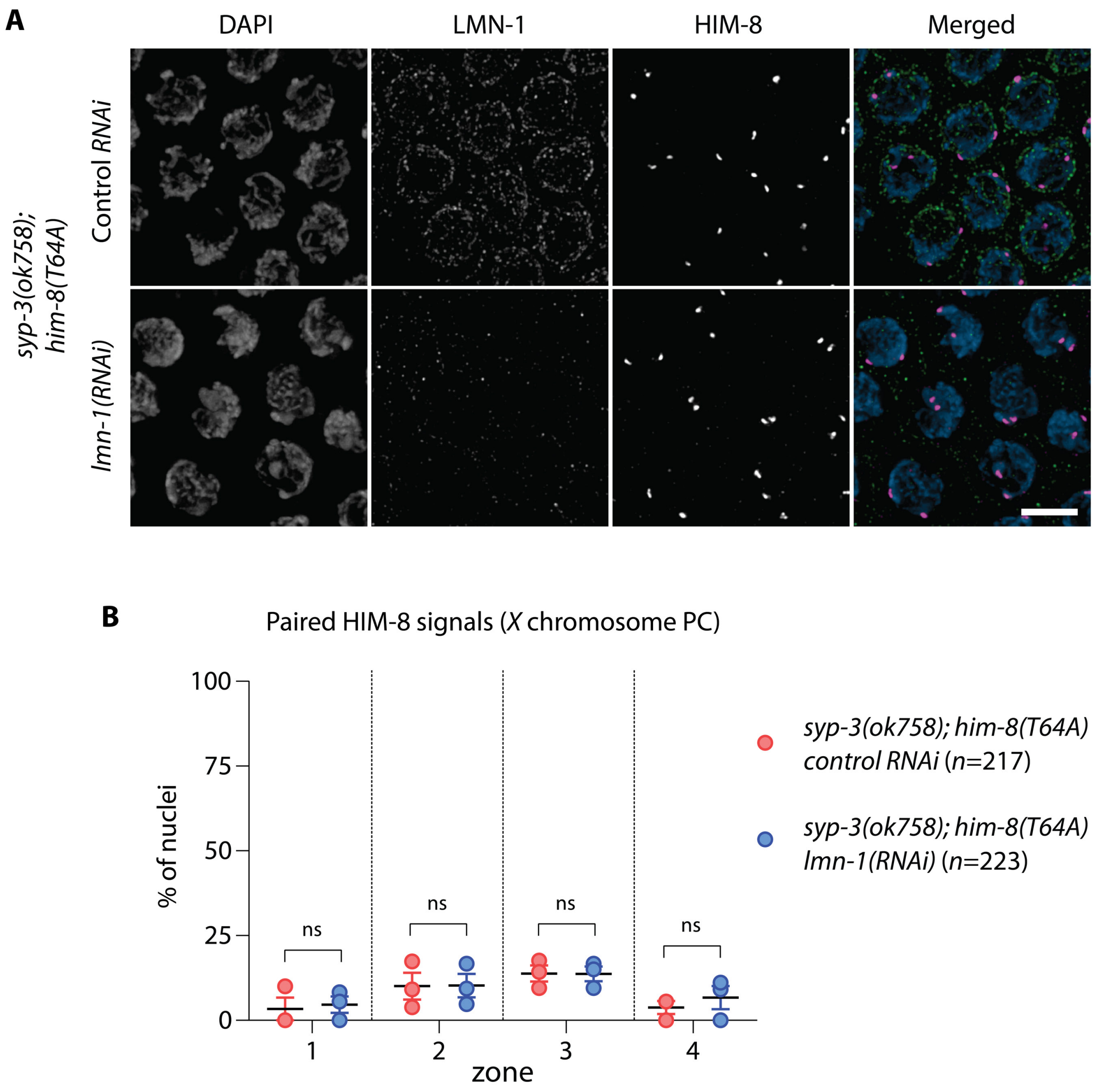

Fig. S10. Depletion of LMN-1 by RNAi fails to rescue pairing in HIM-8^{T64A}. (A), Maximum-intensity projection images of transition zone-pachytene nuclei in *syp-3(ok758); him-8(T64A)* hermaphrodites, either following 48 hr depletion of LMN-1 by RNAi or control RNAi. Nuclei are stained with antibodies against LMN-1 (green) and HIM-8 (magenta). Scale Bar, 5 µm. **(B),** Quantification of *X* chromosome pairing. The extended transition zone was divided into zones 2-4, with zone 1 corresponding to the pre-meiotic region. Each point represents a single gonad. *p*-values were computed using Student's *t*-test with Bonferroni *post-hoc* correction.

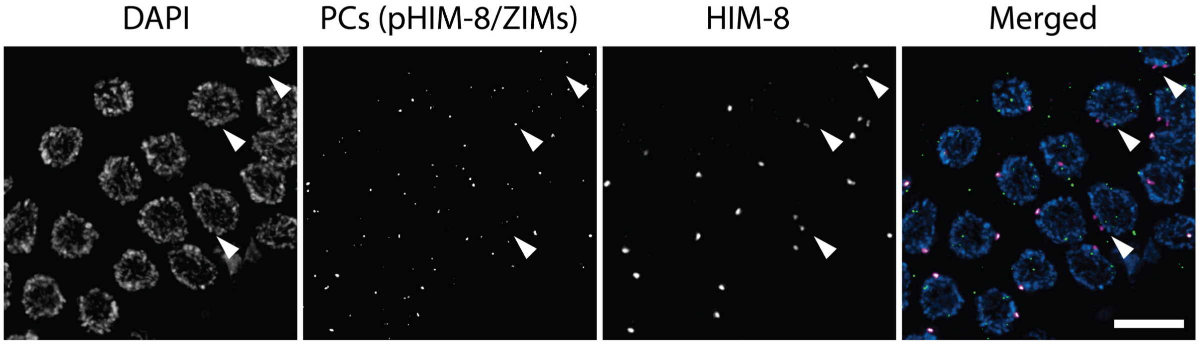

Fig. S11. Loss of PLK-2 from pairing centers occurs concomitantly with dissociation of synapsis-independent pairing. Images show maximum-intensity projections of the proximal region of the gonad, corresponding to the end of the extended transition zone, in *syp-2(ok307)* hermaphrodites. Gonads were stained with antibodies against phosphorylated PC proteins (green) and HIM-8 (magenta). Separation of HIM-8 foci correlates with a loss of phosphorylation of HIM-8, indicative of loss of PLK-2 from the *X* chromosome PC.

Table. S1. crRNA, repair template, and primers used in this study as DNA sequences.

Strain	crRNA (20 nt)	Repair template	Primer pair for genotyping
ha::mjl-1	ACACTTGATTGATTGATTGG	TAATCGAAAGAGATTTTTCTAGCAACTCCTCTTGTATTATATTTCCTAAAATGTACCCATACGATGTCCCAGATTACGCTTACCCATACGATGTCCCAGATTACGCCGGAGGAGGAGGATCCGTTCTTCCAACTGAAATTCCACCAATCAATCAATCAAGTGTTCTAATTGATCTCAAAAAGCGAACAACTAGCTCAATA	TCTCGTCCCGACAGACAGT<br><br>ACAGTGTGGTGAGGGCAATC
gfp11:v5::mjl-1	ACACTTGATTGATTGATTGG	TTTCTAGCAACTCCTCTTGTATTATATTTCCTAAAATGCGTGATCACATGGTTCTTCACGAGTACGTCAACGCTGCTGGAATCACCGGAGGATCCGGAGGATCCGGAAAGCCAATCCCAAACCCACTCCTCGGACTCGACTCCACCGGAGGAGGATCCGGATCCGTTCTTCCAACTGAAATTCCACCAATAAATCAATC	ACAACTAGCTCAATAGAAGCCGT<br><br>CGCTTACTGATGCATCTGAATAAT
mjl-1(Δacidic)	CTGTAAAATTTCGACATTAA<br><br>ATTAACATACAGCTCAATTT		CTTCGATGAAGAAGGAAATGTGGTATGACTGGC<br><br>CTCTTCGATTGCCGACTCTTTCCATCCTTT
mjl-1(ie142)			CTTCCATTGCCTCGACCTGA<br><br>TGAACAAGGAGCTAGCCGAC
mjl-1(tm1651)			TCTCGTCCCGACAGACAGT<br><br>ACAGTGTGGTGAGGGCAATC
aid::v5::sun-1			TTGTAAACTCTACCAGCCT<br><br>TCAGAGGTAGTTTAGTGGC
aid::ha::zyg-12			TGAACACCGCATTCTACGAAC

			CAATAGAATCTGTG ACCCCTGCT
